# Perceptions of medical students towards healthcare devolution: an online cross-sectional study

**DOI:** 10.11604/pamj.2015.20.355.4714

**Published:** 2015-04-14

**Authors:** Henry Nyongesa, Cecilia Munguti, Christopher Odok, Winstar Mokua

**Affiliations:** 1 University of Nairobi, School of Medicine, School of Medicine, Nairobi, Kenya

**Keywords:** Medical students, healthcare, devolution, perceptions, online

## Abstract

**Introduction:**

There have been worries concerning the preparedness and capacity of the counties to take over health care services. As the current medical students are going into this new system, we sought their opinions on the issue of devolution. The objective is to assess beliefs and attitudes of medical students towards devolution of healthcare services.

**Methods:**

A cross sectional survey was conducted at University of Nairobi medical school during the period of February-May 2014. Though a calculated random sample of 384 medical students was powerful enough to fulfill our objectives, all eligible medical students were invited by email to fill in a semi structured online questionnaire. Computed results from Google sheets were reported in frequencies and percentages.

**Results:**

Data was collected from 191 respondents with majority of them in their clinical years (levels 3, 4 and 5) of study. More participants considered working in private/ mission health institution (40%) after graduating as compared to public or non health institution (30%). The media provided most of information concerning devolution (77%). Few respondents reported using government documents (36%) or public forums (24%) to get information on healthcare devolution. While most of the respondents were of the opinion that health information system (68%), health finance (63%), procurement of medical products (54%), leadership and governance (73) should be devolved, only 18% wanted health personnel to be devolved. Most of the opinions on healthcare devolution were not in agreement with the goal of devolution: more than 50% thought the process would not result in improved efficiency, resource allocation, disease control programs or maintenance of infrastructure.

**Conclusion:**

Despite the envisioned benefits of healthcare devolution, there is a low opinion among medical trainees concerning these reforms and their implementation. Nevertheless, it is early to speculate whether such viewpoints will be carried to the future once teething problems are dealt with.

## Introduction

Devolution of services is a broad continuum of events that culminates in transfer of power, authority and decision making powers to the local authorities [1]. In countries where devolution has been managed well, there is reported increase in access, utilization and management of the health services [1]. There is also availability of adequate health workforce, drugs, medical equipment and timely attendance to patients needs.

Despite elaborate implementation of devolved health services in some countries, there are existent challenges pertaining to a number of issues. Key among these concerns is expenditure devolution [2, 3]. Inequity and delays in disbursement to the various units with favoritism to politically correct areas may also limit health care availability and utilization [4]. To adequately fund health care, the local authorities usually undertake an extraneous way of generating funds. An allocation formula for health finances that is stringently followed is drawn by independent parties factoring in the population size and geographic size [5].

Furthermore, healthcare personnel tend to concentrate in major urban areas hence denying services to far flung local authorities [6, 7]. Employing highly skilled specialists to such areas may prove very difficult. There is also likelihood of poor remuneration especially where the payroll is determined at the local level which may consequently lead to reduced performance. Institutional weaknesses can result in recruitment of unqualified staff to satisfy political interests or nepotistic tendencies. As a solution to these challenges, there has been a tendency to recentralize the major skilled workers with low cadre workers being recruited and paid by the local governments [6]. Another model applied is the contractual bonding where health care workers are deployed in a remote area for a given period of time with additional financial payoff [8, 9].

In the Kenyan scenario, devolution of health care services to the 47 county governments is heavily driven by constitutional pressures. Whether devolution is the panacea to challenges facing public healthcare or not has not been described in any substantive paper. Yet, this represents one of the radical healthcare reforms in the post colonial period. In brief, health legislative process is within the domain of central government with ultimate decision making and implementation being vested upon counties. In principle, therefore, the ministry of health is comparatively weaker considering shared responsibilities with other ministries and decisive role of regional health ministries. In these initial stages, health services from the lowest delivery point (health centre) to provincial district referral hospitals have been put under the management of county governments. To expedite the process, county government health docket is run by county director of health. The county public service commission, on the other hand, is mandated to recruit, discipline and dismiss the personnel.

Although the government has instituted a raft of measures to hasten the process, there are multiple issues that have not been addressed. The procurement policies, infrastructural development and health care personnel recruitment, their wages and working conditions are among pertinent issues. There is no standard framework concerning employment, deployment, transfer and remuneration of healthcare personnel with each county purporting to come up with county tailored policies. So longer as these issues exist, the public discourse on merits and demerits of devolution will continue being elicited.

Ultimately, whether healthcare devolution will succeed or not is heavily dependent on how central and local government agencies treat the process. Though public opinion is tilted towards full devolution of services, what concerns current healthcare trainee is the uncertain future of public healthcare. Currently, there is little evaluative information concerning the process, making speculation to be rife. From historical times when the government used to post medical graduates for remunerated internships and work in various health institutions across the country, the trend is likely to change in the new dispensation with the immense powers vested upon the county governments. This study aimed to seek opinions among medical trainees concerning the process albeit even after implementing through constitutional referendum.

## Methods

### Study design

The current study was a cross-sectional online survey undertaken from February 2014 to May 2014 using a questionnaire for data collection from medical students at undergraduate levels of study. The questionnaire constructed using *Google form*s, was emailed to eligible participants.

### Study population

This study targeted medical students undertaking their studies at University of Nairobi during the study period. The university has an estimated population of 1500 undergraduate medical students distributed almost equally in the five levels of study. Since most students access posted notes via their email addresses, it was presumed that they could as well access other non academic content posted via email if informed of the purpose. Students who did not have email addresses or were not affiliated to the university but undertaking their elective sessions within the medical school was not eligible for study.

To calculate a sample size, the following factors were considered: margin of error (5%), response rate (60%) and the anecdotal prevalence (70%). The estimated sample size arrived at 384 after using the sample size calculator (Creative Research Systems in Epi Info 7). But since there was minimal experience with online questionnaires, there was a possibility of high non response despite measures to minimize. We therefore administered the questionnaire to all students who had emails in the respective class emails’ contacts. To maximize response rate, a verbal request and a clear explanation on the importance of the study shall be made in various classes. The process of accessing, filling in and submitting the questionnaire was explained in each class by the research coordinators. No incentives were offered for filling and submitting the questionnaire. All the students recruited in the study were assured of confidentiality, anonymity and the principle of voluntary participation. This was further reinforced by Google requirements highlighted at the bottom of every form and anonymity on submission of the forms. Any issues concerning the questionnaire were clarified by members of the research team whose email addresses were availed on the form. There was no follow up after the participants had exited the survey.

### Research instrument

The questionnaire used for data collection was arrived at after relevant literature search and plenary discussion among the members of the research team. Emerging topics on devolution of healthcare were developed followed by literature search. A plenary discussion was then held where each member presented the findings. Information obtained was collated and proof read by members before designing an online questionnaire using Google forms. This was later pre tested on a convenience sample of 25 nursing students. Four domains were assessed in the questionnaire. Firstly, demographic profile to find out the student's level of study, gender, residence (whether urban or rural) and preferred institution of work (public or private). The next section determined level of knowledge and sources of information for devolution in Kenya. This was followed by a Yes/No/No opinion assessment of beliefs concerning the devolution. Ten questions were presented in which the respondent was required to choose the level of agreement to the statements.

### Statistical analysis

Descriptive data expressed as sums and percentages of all variables across all participants was computed from a linked Google sheets.

### Ethical approval

Ethical consent for the study was given by the University of Nairobi/Kenyatta National Hospital Ethical Review Board after submitting the full details of the study protocol.

## Results

Information was collected from 191 students representing response rate of 50%. Table 1 summarizes the basic profile of the respondents. Majority of them were from the clinical years. Three quarters (143) of the respondents said they live in urban areas. More respondents (40%) would prefer working in a private or mission hospital than work in public or non health institution.

**Table 1:** Demographic profile of respondents (N = 191)

Aspect	Variable	Frequency	Percent
Gender	Male	98	51
	Female	93	49
Level of study	3	74	39
	4	65	34
	5	52	27
Place of residence	Urban	143	75
	Rural	48	25
Place of preference	Public health institution	58	30
	Private/mission institution	76	40
	Non health institution	57	30

**Figure 1:**
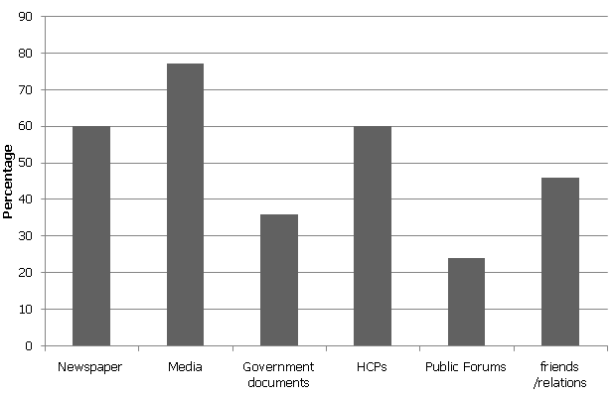
Sources of information on devolution (N = 191)

Figure 1 shows the sources of information that respondents used to acquire knowledge on the issue of healthcare devolution. The commonest source of information was media (radio and television) (77%), followed by newspapers and healthcare professionals (HCPs) (60% each). Least commonly used sources were government documents (36%) and public forums (24%).

**Figure 2:**
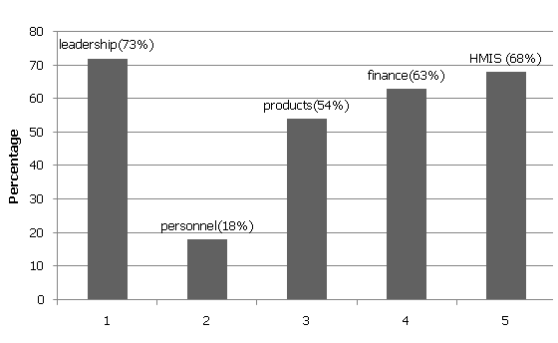
Devolution of health system building pillars (N = 191)

When asked about what aspect they wanted to be devolved, more than half of the respondents cited heath information management system (HMIS) (68%), finance (63%), leadership (73%) and medical products (54%). Notably, only 18% respondents suggested devolution of healthcare personnel to the counties (Figure 2).

To seek opinion on the impact of devolution on healthcare, we asked several questions. Less than half of the respondents were of the opinion that devolution will bring services closer to people (27%), develop centres of excellence (35%), enhance resource allocation to the counties (46%) and increase disease control programs (32%). In the same breath, more than half thought that devolution will result in decline in efficiency and effectiveness (73%), promote local political interference (90%), reduce healthcare funding (63%), lead to decline in infrastructure (58%) and public utilization of public health facilities (52%) (Table 2).

**Table 2:** Opinions on impact of devolution on health care (N = 191)

Opinion	No (%)	Yes (%)	No opinion (%)
It will bring citizens closer to decision making	122 (64)	51(27)	18(9)
It will enhance resource allocation to the counties	97(51)	88(46)	6(3)
Lower efficiency and effectiveness	44(23)	139(73)	8(4)
It will increase coverage of disease control programs	113(59)	61(32)	17(9)
Development of centres of excellence	120(63)	67(35)	4(2)
Underfunding of health care	46(24)	120(63)	25(13)
Increase local political interference	17(9)	172(90)	2(1)
Poor staff morale	21(11)	168(88)	2(1)
It will decrease utilization of public health services	61(32)	111(58)	19(10)
Decline in maintenance of infrastructure	82(43)	99(52)	10(5)

## Discussion

Our findings on perception of medical trainees towards devolution of healthcare concur with other studies that have reported on this matter [10]. There is clearly an apathetic attitude towards the devolution process from the students’ standpoint. Despite the conceptualized benefits as outlined in the constitution, most of the trainees’ opinions are diametrically opposite.

For instance, while the devolution principle is to foster equity and equality in resource allocation, service delivery and develop county referral hospitals as centres of excellence [11, 12], the students opinion is tilted towards the negative. And hence when devolution is mentioned in association with healthcare underfunding, poor staff morale, decreased public healthcare utilization, increased political interference, the affirmation is overwhelming. This negative response to devolution is perhaps ascribed to many years of institutional and personnel neglect by the government. In theory and sometimes in practice, the relevant ministries have come up with all manner of documents meant to foster efficiency in service delivery. The impact has been dismal.

In this study, we reported a great majority of participants having a low opinion of the current state of public healthcare in their counties. We hypothesize that at the center of this sorry state of public health is the unsupportive political climate that has treated health care with wanton alacrity. Given the usual political meddling in matters to do with health, it was not surprising that our respondents ranked the facilities lowly. This finding is not a case in isolation considering findings from other studies that have noted the mismatch between medical professionals and political environment [13–16].

Although we noted that most of the participants were well versed with devolution from media and fellow colleagues, the admission that few of them used government documents or public forums for sourcing information may have limited their full understanding of devolution. But this is not surprising even in advanced countries where HCPs rarely focus on government gazettes [17]. The taxing nature of medical training curriculum may also have not allowed them to acquire extraneous knowledge from the two channels. These two channels are at the crux in detailing the steps, policies and implementation process of devolution. Hence lack of knowledge from these two places may have placed the respondents at a disadvantaged position in commenting on devolution. The reliance on a sensational media that highlights the government in the negative may be a stimulus for the given responses.

In light of unfruitful industrial action by healthcare professionals against devolution of health care services two months prior to conducting this study, it was not surprising to obtain such results. We postulate that the strike may have had an impact on the psychological mindset of our respondents, just to be in harmony with their senior colleagues.

There are a number of limitations worth considering when interpreting this study. Firstly, as has been described, there might have been bias in responses when the study was conducted after a healthcare workers’ strike. Initially, we had set out to interview all levels of education in medical school. However, due to logistical reasons (the first two levels being out of session), we were not able to assess them. Nonetheless, we believe that our results reflect the situation for the 3 coming generations of doctors produced by this university.

## Conclusion

There is a low opinion of medical students towards health care devolution. Nevertheless, it is early to speculate whether such viewpoints will be carried to the future once they familiarize themselves with devolution content. At the moment, there are myriad teething problems affecting devolution in entirety. With positive results coming from a few counties and a potential snowball effect, it will not be amazing to see these viewpoints tilted towards devolution.
